# *Oncomelania hupensis* Distribution and Schistosomiasis Transmission Risk in Different Environments under Field Conditions

**DOI:** 10.3390/tropicalmed8050242

**Published:** 2023-04-23

**Authors:** Yinlong Li, Suying Guo, Hui Dang, Lijuan Zhang, Jing Xu, Shizhu Li

**Affiliations:** National Institute of Parasitic Disease, Chinese Center for Disease Control and Prevention (Chinese Center for Tropical Disease Research), NHC Key Laboratory of Parasite and Vector Biology, WHO Collaborating Center for Tropical Disease, National Center for International Research on Tropical Disease, Shanghai 200025, China

**Keywords:** *Oncomelania hupensis* snails, schistosomiasis, surveillance, risk of schistosomiasis transmission

## Abstract

The goal of schistosomiasis prevention and control in China is shifting from transmission interruption to elimination. However, the area inhabited by the intermediate host, the snail *Oncomelania hupensis*, has not changed much in recent years. Different environmental types have different impacts on snail breeding, and understanding these differences is conducive to improving the efficiency of snail monitoring and control and to saving resources. Based on previous epidemiological data, we selected 199 villages in 2020 and 269 villages in 2021 from transmission control, transmission interruption, and elimination areas of snail breeding. Snail surveys were conducted in selected villages using systematic sampling and/or environmental sampling methods in six types of snail-breeding environments (canals, ponds, paddy fields, dry lands, bottomlands, and undefined environments). All live snails collected from the field were evaluated for *Schistosoma japonicum* infection using the microscopic dissection method, and a subsample of snails was subjected to loop-mediated isothermal amplification (LAMP) to assess the presence of *S. japonicum* infection. Snail distribution data and infection rate and nucleic acid positive rate of schistosomes in snails were calculated and analyzed. The 2-year survey covered 29,493 ha of the environment, in which 12,313 ha of snail habitats were detected. In total, 51.16 ha of new snail habitats and 107.76 ha of re-emergent snail habitats were identified during the survey. The occurrence rate of snails in canals (10.04%, 95% CI: 9.88–10.20%) and undefined environments (20.66%, 95% CI: 19.64–21.67%) was relatively high in 2020, and the density of snails in bottomlands (0.39, 95% CI: 0.28–0.50) and undefined environments (0.43, 95% CI: 0.14–1.60) was relatively high in 2021. Of the 227,355 live snails collected in this study, none were *S. japonicum*-positive as determined by microscopy. Of the 20,131 pooled samples, however, 5 were *S. japonicum*-positive based on LAMP analysis, and they were distributed in three environmental types: 3 in bottomland, 1 in dry land, and 1 in a canal. The bottomland environment has a high risk of schistosomiasis transmission because it contains a large area of newly emerging and re-emerging snail habitats, and it also had the most breeding snails infected with *S. japonicum*. Thus, this habitat type should be the key target for snail monitoring and early warning and for the prevention and control of schistosomiasis.

## 1. Introduction

Schistosomiasis is a zoonotic disease caused by the parasitic trematode worm *Schistosoma japonicum*, which is transmitted through various means. In China, this disease poses a serious public health threat, and its transmission is largely dependent on the freshwater snail *Oncomelania hupensis*, which serves as the sole intermediate host for *S. japonicum* [1,2]. Despite this challenge, China has made significant progress in controlling and preventing schistosomiasis over the last 70 years. In fact, the implementation of an integrated control strategy in the early 2000s [3] has led to a substantial reduction in both the prevalence and intensity of *S. japonicum* infections [4]. Currently, the epidemic status of schistosomiasis is at a low level, and the infection rate of people has decreased significantly from 2.04% in 2005 to 0.000045% in 2021 [5].

However, the area inhabited by *O. hupensis* has remained at about 3.5 billion m^2^ since 2004, and newly emerging and re-emerging snail habitats have been reported in many areas [1,6]. Thus, this intermediate host [7] has a wide distribution range in endemic areas of China. As the flow of people into schistosomiasis-endemic areas increases, it is still possible for *S. japonicum* to complete its life cycle in the wild [8]. Existing research shows that traditional anatomical microscopy methods for the detection of *S. japonicum* infection in snails can miss its presence, which increases the risk of schistosomiasis transmission [9]. Researchers have predicted that new occurrence and recurrence of snails are most likely to occur in Poyang Lake, Dongting Lake, and the lower reaches of the Yangtze River [10].

Due to the wide distribution of snails and the variety of snail-breeding environments, it is increasingly important to identify the risks in each environment, implement targeted prevention and control measures, and improve the level of monitoring and early warning. This approach will lay a solid foundation for shifting schistosomiasis control in China from transmission interruption to elimination. The goals of this study were to evaluate the distribution of *O. hupensis*, detect *S. japonicum* infection of snails in different environments in the field, and map newly emerging and re-emerging snail habitats. We studied *O. hupensis* for two consecutive years from 2020 to 2021 in schistosomiasis-endemic areas, including transmission control, transmission interruption, and elimination areas where snails breed.

## 2. Materials and Methods

### 2.1. Study Sites

Based on epidemiological data from previous years, we selected one administrative village from each endemic county with expected potential risk of schistosomiasis transmission. In 2020 and 2021, 199 and 269 villages, respectively, were selected. The villages mentioned above shared at least one of the following characteristics: (1) previously had a high prevalence of schistosomiasis, and the snail habitats in their environment remained essentially unchanged; (2) exhibited an increase in breeding areas or density of living snails, detected infected snails or the emergence of new snail habitats, and had a relatively high rate of infection or positivity for antibodies in humans or livestock according to the latest survey; (3) experienced significant changes in their snail-breeding habitats due to natural disasters, such as floods or earthquakes, or the construction of large-scale water conservancy, transportation, and other projects; or (4) underwent large-scale population migration or movement, which increased the likelihood of schistosomiasis occurrence.

Each village was to select at least 5 snail environments (all of which were insufficient) and/or suspicious environments, and the total number of snail inspection frames in each risk monitoring village was not to be less than 200 frames, and all snails in the frame were to be captured. In the spring of each year, systematic sampling and/or environmental sampling methods were used to investigate snails in the environment with snails and/or suspicious environment, and the longitude and latitude of each environment were recorded. Crushing microscopy was used to detect *S. japonicum* in all snails collected (dead and alive), and loop-mediated isothermal amplification (LAMP) was used to detect at least 500 live snails (all of which were insufficient). In order to improve the monitoring efficiency of the risk environment, the number of live snails detected by LAMP in each snail environment was not to be less than 100 (all the insufficient ones were to be performed, and the results were to be recorded.

### 2.2. Snail Surveys

We conducted the snail survey in each village in the spring of 2020 and 2021 using systematic sampling and/or environmental sampling methods.

#### 2.2.1. Systematic Sampling

For environments where snails are abundant and scattered, we adopted a systematic sampling survey method. The specific process was to set up a checkpoint at a certain distance and check one frame at each point, with a frame size of 0.1 m^2^ (31.7 cm × 31.7 cm). The interval distance between checkpoints was determined based on the size and length of the environment at that time. We adopted the method of inspecting every 5 m and 10 m for river ditches and ponds. To conduct snail surveys on rivers and lakes, beaches, fields, and other areas, we adopted a vertical and horizontal systematic sampling (i.e., chessboard sampling) survey method. We set several parallel inspection lines on the beaches and fields, then set inspection points (boxes) along each inspection line at equal distances. For the distance between lines and the distance between points on each line, we used a space of 5 to 20 m based on the size of the beaches and fields. We increased the distance length for environments for large beaches, but none exceeded 50 m. We divided it into several blocks in an environment with an extensive beach area. Then, we conducted a vertical and horizontal systematic sampling survey within each block so that the distribution of checkpoints evenly penetrated the entire survey area. We captured all the snails at the inspection point and registered the location, environment type, number of snails, and other relevant information. Then, in the laboratory, we observed the survival rate, detected the positive rate of snails, and recorded the occurrence rate of positive snail frames.

#### 2.2.2. Systematic Sampling Combined with Environmental Sampling

During the survey, we set up a systematic sampling survey with a frame every 5 m or 10 m equidistant for snail-breeding environments, such as rivers, ditches, ponds, and ridges. If we did not detect snails at two adjacent points (frames), we selected an environment where snails are easy to breed in the interval between the two points (frames) and randomly inspected two points (frames) simultaneously. We used systematic sampling combined with environmental sampling to calculate the area with snails, which extended 20 m from the left to the right. For a large beach, we first divided it into several blocks, each with an area of about 133,340 m^2^. Then, within each block, we used a vertical and horizontal system to select points (frames), generally 100 to 200 points (frames) per area; if we did not detect snails at four adjacent points (frames) in the vertical and horizontal system sampling, we searched for four points (frames) in the area between the four points where snails are prone to breeding.

We surveyed various environments, including canals, ponds, paddy fields, dry lands, bottomlands, and undefined areas. To count snails, we used a 0.1 m^2^ frame. Based on the previous survey, we employed a systematic sampling approach in environments containing breeding snails. In neighboring habitats, we conducted environmental sampling first and then systematic sampling when we found living snails. We determined the distance between any two frames and the number of frames used based on the ecological characteristics of the surveyed environments. Subsequently, we tallied and collected all snails within the frames and brought them to the laboratory for examination.

### 2.3. Microscopic Dissection of Snails

**Figure 1:**
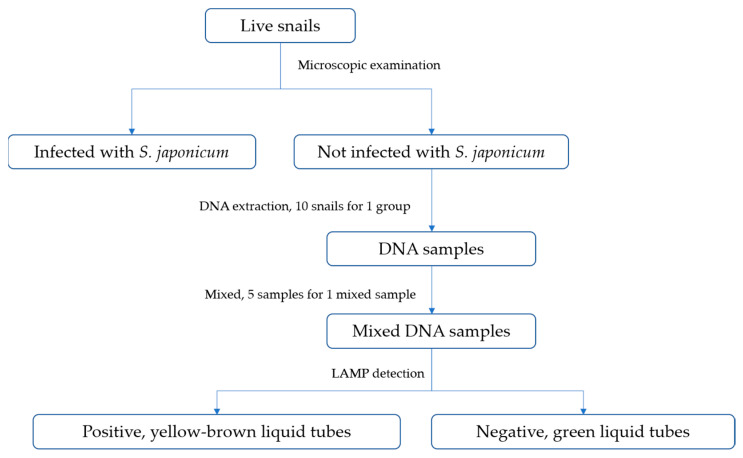
A flowchart with steps of sample analysis.

After identifying and washing the *O. hupensis* snails based on their shell morphology, we placed them in a dish filled with dechlorinated water at room temperature for 2–3 h. Any snail showing signs of movement, extension of soft tissue, or contraction after being probed was considered alive. The living snails were gently crushed using another thick slide and examined under a microscope after adding a drop of dechlorinated water. To determine if a snail was infected with *S. japonicum*, we examined its soft tissue under a microscope and looked for sporocysts and/or cercariae. If we observed sporocysts and/or cercariae of *S. japonicum* in the snail’s soft tissue, we considered it infected (Figure 1).

### 2.4. Sample Analysis

#### 2.4.1. DNA Extraction

We used the TIANamp Genomic DNA Kit (TIANGEN BIOTECH (BEIJING) CO., Ltd., Beijing, China) and followed the manufacturer’s protocol and obtain the required DNA from snails using this DNA extraction kit. Groups of five tubes of extracted DNA from the same environment were pooled into one sample for loop-mediated isothermal amplification (LAMP) detection.

#### 2.4.2. LAMP Detection

The DNA of *S. japonicum* was detected by using genomic DNA extracted from pooled snail samples as a template for the LAMP assay. The primers targeting the mitochondrial 28S rRNA gene of *S. japonicum* were used in the reaction, which was carried out in a final volume of 25 µL. The primer sequences were: F3 (5′-GCTTTGTCCTTCGGGCATTA-3′), B3 (5′-GGTTTCGTAACGCCCAATGA-3′), FIP (5′-ACGCAACTGCCAACGTGACATACTGGTCGGCTTGTTACTAGC-3′), and BIP (5′-TGGTAGACGATCCACCTGACCCCTCGCGCACATGTTAAACTC-3′).

The volume of each component was 2.0 µL (genomic DNA template), 12.5 µL (2 × LAMP reaction buffer), 1.0 µL (amplification primer mixture), 7.5 µL (sterilized deionized water), 1.0 µL (Bst DNA polymerase), and 1.0 µL (chromogenic reagent (New England Biolabs).

Positive and negative controls were included in each amplification reaction, where genomic DNA extracted from adult *S. japonicum* and sterilized deionized water were used to replace the extracted DNA template, respectively. After incubating the reaction tubes at 65 °C for 60–90 min, we observed visually without the need for any additional instrumentation or analysis. Tubes with green liquid indicated the presence of *S. japonicum* DNA and were considered positive, while the remaining tubes with yellow-brown liquid were negative.

### 2.5. Map of Geographical Distribution of Snails

All selected villages were uniformly numbered and located in longitude and latitude. We collected the area of new and recurrent snails corresponding to each geographical location and built a database from 2020 to 2021. We then imported the database in to ArcGIS 10.1 software to generate a map of the distribution of snails in traditional and newly developed or re-emerging snail habitats

### 2.6. Data Management and Analysis

We recorded annual data and organized it in a Microsoft Excel spreadsheet. We calculated the snail distribution area by multiplying the length (m) and width (m) of the surveyed environments. The dimensions were based on the furthest distance between two frames where either live or infected snails were found. To determine statistical significance, we calculated confidence intervals (CIs) using standard formulas based on the binomial distribution.

## 3. Results

### 3.1. General Information about Selected Study Sites

We first selected all the transmission control counties and at least 20% of the counties with snails (including transmission interruption counties and elimination counties). We then selected some villages in these selected counties to survey snail breeding in the field in areas with different environmental types. Ultimately, we surveyed 199 villages in 2020 and 269 villages in 2021.

### 3.2. Distribution of Snails in Different Environments

We conducted this investigation in an area covering 29,492.81 ha. Among the 12,312.92 ha confirmed to contain breeding snails, 51.16 ha were newly developed snail habitats, including canals (2623 ha), ponds (247.23 ha), paddy fields (2416.77 ha), dry lands (1073.31 ha), bottomlands (22,409.44 ha), and undefined environments (723.06 ha). Additionally, 1072.76 ha were re-emergent snail habitats, including canals (952.26 ha), ponds (79.64 ha), paddy fields (300.98 ha), dry lands (150.12 ha), bottomlands (10,530.67 ha), and undefined environments (299.22 ha).

During the snail investigation in 2020–2021, we investigated 931,735 frames and discovered 227,355 living snails in 67,798 frames. The percentage of frames containing living snails varied between 6.88% (95% CI, 6.81–6.95%) and 7.69% (95% CI, 7.61–7.77%). Furthermore, the average live snail density ranged from 0.19 (95% CI, 0.19–0.28) in 2020 to 0.3 (95% CI, 0.24–0.31) (Table 1).

**Figure 2:**
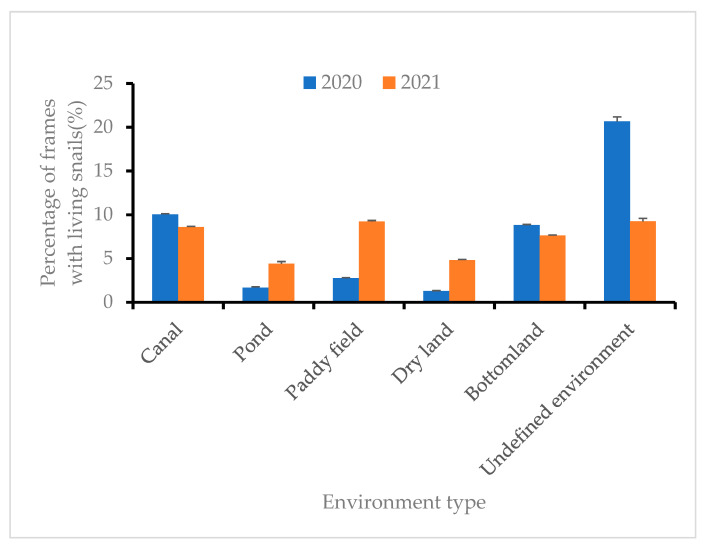
Percentages of frames containing live snails in the six different environment types in 2020 and 2021. Six different environment types: Canal, Pond, Paddy field, Dry land, Bottomland, and Undefined environment. Among the six environments, the percentage of frames containing living snails of Undefined environments in 2020 is higher than that of the other five environment types. In 2021, the occurrence rate of live snail frames in the four environments (Canal, Paddy field, Bottomland, and Undefined environment) is close to and higher than that in the remaining two environments (Pond and Dry land).

The percentages of frames containing living snails in the undefined and canal environments were high (10.04%, 95% CI: 9.88–10.20% and 20.66%, 95% CI: 19.64–21.67%, respectively) in 2020, while the values in pond and dry land environments were low (1.66%, 95% CI: 1.43–1.90% and 1.28%, 95% CI: 1.20–1.40%, respectively in 2020) (Figure 2).

**Figure 3:**
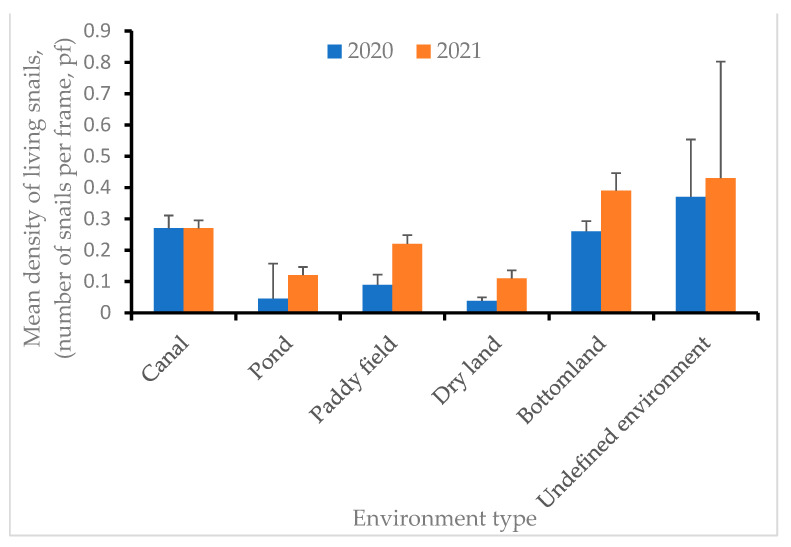
Mean densities of living snails in the six different environment types in 2020 and 2021. Six different environment types: Canal, Pond, Paddy field, Dry land, Bottomland, and Undefined environment. In addition to canals, the average density of live snails in other environments in 2021 is higher than in 2020. Among the six environments, the average live snail density of Undefined environments in the same year is higher than that of the other five environment types.

The average density of snails varied among the different environments. The mean densities of snails in bottomland and undefined environments were relatively high (0.39, 95% CI: 0.28–0.50 and 0.43, 95% CI: 0.14–1.60, respectively) in 2021, whereas densities in pond and dry land environments were low (0.045, 95% CI: 0.00–0.44 and 0.038, 95% CI: 0.014–0.059, respectively) in 2020 (Figure 3).

### 3.3. Presence of S. japonicum in the Snails

From 2020 to 2021, 227,355 living snails were analyzed using the dissection method, but no *S. japonicum*-positive snails were detected by microscopy. We randomly selected soft tissue from 134,302 dissected snails, which accounted for 59.07% of the total snail population. Then, we extracted DNA from these samples to obtain 20,131 mixed nucleic acid samples. Among those mixed samples, only five were positive for *S. japonicum* in the LAMP analysis. Three of the positive nucleic acid-mixed samples were found in a bottomland environment (3/5, 60%), one was found on a dry land environment (1/5, 20%), and one was found in a canal (1/5, 20%) environment. The mean positive rate of mixed nucleic acid samples was 0.018% (95% CI: 0.00–0.039%) in 2020 and 0.059% (95% CI: 0.00–0.13%) in 2021 (Table 2).

### 3.4. Geographical Distribution of Newly Developed and Re-Emergent Snail Habitats

**Figure 4:**
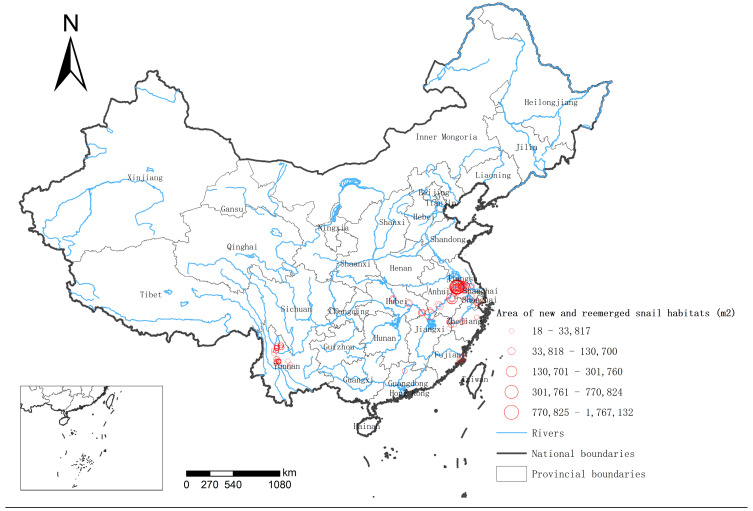
Geographical distribution of newly developed snail habitats and re-emergent snail habitats in 2020 and 2021. The size of the red circle is positively correlated with the snail area it represents.

The geographical distribution map showed that newly developed snail habitats and re-emergent snail habitats were mainly located in Poyang Lake, the lower reaches of the Yangtze River, and some of Yunnan’s mountainous areas in 2020 and 2021. The scale of the single environments in which newly developed snail habitats or re-emergent snail habitats were found ranged from 18 m^2^ to 1,767,132 m^2^ (Figure 4).

## 4. Discussion

China has made significant progress in controlling schistosomiasis through sustained efforts over the past few decades [11], and the goal is to eliminate the disease in all counties by 2030 [12]. However, the goal of removing schistosomiasis in all counties by 2030 is challenging due to the zoonotic transmission of *S. japonicum* and the fact that snails, the intermediate host, can live both in water and on land [13,14,15]. Therefore, to ensure effective control and elimination of schistosomiasis, it is essential to enhance surveillance measures and use the information obtained to guide the implementation of appropriate strategies. By strengthening surveillance efforts, we can gather crucial data to help us better understand the epidemiology of the disease and develop targeted interventions to control its spread.

*O. hupensis* is the only intermediate host of *S. japonicum*. Although the area inhabited by these snails has not changed much since 2004, in some endemic areas, it has shown an upward trend [10]. We found that newly developed and re-emergent snail habitats are mainly located in the lower reaches of the Yangtze River and the Poyang Lake area, which may be related to floods in recent years and seedling transplantation in epidemic areas [16,17].

It is crucial to comprehend the distribution of its vector, *O. hupensis*, and its infection status as a vector-borne disease to evaluate the prevalence and transmission of schistosomiasis [18,19]. Snails can breed in different types of environments, and environment affects the density, occurrence rate, and area of newly emerging and re-emerging snails. Therefore, the risk index for *S. japonicum* transmission differs among environments. The detection of *S. japonicum* nucleic acids in tissues of three samples based on the LAMP method revealed that the risk of disease transmission was higher in the bottomland environment than in the other five environment types. However, one *S. japonicum*-positive sample was also found in the dry land environment, which had the highest positive rate among all environment types (0.59%; 95% CI: 0.0–1.8) in 2020, and in the canal environment, with the second-highest average positive rate (0.093%, (95% CI: 0.00–0.28).

Newly developed and re-emergent snail habitats are still a big challenge in endemic areas [20] because *O. hupensis* is the only intermediate host of *S. japonicum*. The effective control of snails, mainly through environmental management, has played a significant role in successfully controlling this parasite in China [21]. *O. hupensis* control is one of the important means to control schistosomiasis and is also one of the important measures in the medium- and long-term planning of schistosomiasis control in China. At the same time, China has been actively exploring methods, such as biological and drug snail control, to combat schistosomiasis. However, due to the highly complex breeding environment of schistosomiasis, the large size of the existing snail population, and restricted access to some snail-breeding areas, it has been challenging to implement effective snail control measures. As a result, the snail-breeding area remains extensive making it difficult to achieve the goal of eradicating snails in the future. In addition, the close communication between people in schistosomiasis-endemic areas, the frequent circulation of goods, and the frequent scattering of livestock in endemic areas make the transmission risk of schistosomiasis japonica still existent. Therefore, studies of the breeding environments of *O. hupensis* are needed for the rational use of resources, control of snails, reduction in the risk of schistosomiasis transmission, and provision of data support for accurate prevention and control of snails as well as for a reference for the elimination of schistosomiasis in the future.

This study has some limitations that should be acknowledged. Firstly, the selection of villages at potential risk of schistosomiasis was not randomized, and due to cost constraints, only one third of the dissected snails were tested using the LAMP method. This may have resulted in a biased estimation of the snail infection status. Secondly, while the LAMP results could identify areas with transmission risk, the pooling strategy used could not provide an accurate estimation of the snail infection rate. Future research should explore modeling methods to estimate infection rates and transmission intensity accurately. Third, the snail-breeding environment is very complex, which makes it difficult to define certain areas as being a single environment type. To address this issue, we used an undefined environment category, which makes the snail survey more comprehensive and accurate.

## 5. Conclusions

The areas of newly developed and recurrent snail habitats are basically consistent with the results predicted by our previous research. Among the six kinds of snail-breeding environments, bottomland areas have a high risk of schistosomiasis transmission because they contain large areas of newly emerging and re-emerging snail habitats and they have the highest proportion of breeding snails infected with *S. japonicum*, as shown by nucleic acid detection. In the future, the monitoring and early warning of snails in bottomland areas should be a top priority for the prevention and control of schistosomiasis.

## Figures and Tables

**Table 1:** Summary of snail surveys in different environments in 2020 and 2021. Note that “ha” means hectare.

Year	Environmental Type	No. Areas Surveyed (ha)	No. Areas with Infested Snails (ha)	No. Areas of New Snail Habitats (ha)	No. Areas with Snails Re-Emerged in Previous Habitats (ha)	No. Frames Surveyed	No. Frames with Snails	No. Living Snails
2020	canal	1168.65	211.13	0	2.9	135,814	13,636	36,976
	pond	46.84	3.17	0	0.73	11,316	188	512
	paddy field	1232.56	110.56	0	9.8	84,513	2335	7494
	dry land	357.78	38.89	0	13.09	75,332	964	2841
	bottomland	8728.78	3332.34	0.03	28	163,052	14,379	42,110
	undefined environment	151.26	71.16	0	13.68	6114	1263	2287
	Total	11,685.86	3767.26	0.03	68.2	476,142	32,765	92,220
2021	canal	1454.35	741.13	15.42	21.99	107,557	9254	29,335
	pond	200.39	76.47	0	0.31	7163	316	898
	paddy field	1184.21	190.42	0.025	37.69	64,522	5957	13,953
	dry land	715.53	111.23	6.23	14.98	59,115	2843	6442
	bottomland	13,680.66	7198.33	29.46	924.09	209,968	15,991	81,413
	undefined environment	571.8	228.06	0	5.51	7269	672	3094
	Total	17,806.95	8545.66	51.13	1004.56	455,594	35033	135,135

**Table 2:** The outcomes of LAMP testing for *S. japonicum* in snails for the years of 2020 and 2021.

Year	Environmental Type	No. Live Snails Detected by LAMP Method	No. Mixed Nucleic Acid Samples (Tubes)	No. Mixed Positive Nucleic Acid Samples	Positive Rate %, (95% CI)
2020	canal	25,305	12,460	0	0
	pond	228	70	0	0
	paddy field	3141	1049	0	0
	dry land	1496	169	1	0.59 (0, 1.8)
	bottomland	23,884	2604	2	0.077 (0, 0.18)
	Undefined environment	2121	198	0	0
	Total	56,175	16,550	3	0.018 (0, 0.039)
2021	canal	23,854	1071	1	0.093 (0, 0.28)
	pond	947	120	0	0
	paddy field	9381	589	0	0
	dry land	4682	281	0	0
	bottomland	37,191	1240	1	0.081 (0, 0.24)
	Undefined environment	2072	280	0	0
	Total	78,127	3581	2	0.059 (0, 0.13)

## Data Availability

The data presented in this study are available upon request from the corresponding author.
